# Association between Sarcopenia and Nutritional Status in Chilean Older People Aged 65 Years and Older

**DOI:** 10.3390/nu14245228

**Published:** 2022-12-08

**Authors:** Mirta Crovetto Mattassi, Camila Henríquez Mella, Lissette Pérez Bocaz

**Affiliations:** Departamento de Salud, Comunidad y Gestión, Facultad de Ciencias de la Salud, Universidad de Playa Ancha, Valparaíso 2340000, Chile

**Keywords:** sarcopenia, muscle strength, nutritional status, ageing, body mass index

## Abstract

In 2018 the European Working Group on Sarcopenia in Older People (EWGSOP2) updated the definition of sarcopenia, with loss of muscle strength being the primary feature. The objective is to describe and associate sarcopenia and nutritional status in a group of Chilean older adults aged 65 years and older. Methods: Descriptive, associative and comparative study, with a cross-sectional design and a quanti-qualitative approach. A number of 155 institutionalized and community-dwelling Chilean older people aged 65 years and older participated in the study (year 2018). Sarcopenia was diagnosed using the algorithm proposed by the EWGSOP2. Muscle strength, muscle quantity and physical performance were assessed; Body Mass Index (BMI) and Mini Nutritional Assessment (MNA) were used. Variables were described according to sex and age groups (65–69; 70–79; and ≥80 years). The association between sarcopenia and nutritional status was studied by the application of multivariate logistic regression analysis with adjustments for age and sex. Results: 49.7% and 5.2% of older adults had probable sarcopenia and severe sarcopenia, respectively; 52.9% and 7% had malnutrition by excess and by deficit, respectively, according to their BMI; and 33.5% had malnutrition according to the MNA. Participants with obesity had 3.2 times more risk of presenting sarcopenia, versus subjects with normal nutritional status (OR: 3.2; 95% CI; 1.24; 8.26). Conclusion: Sarcopenia, as defined by the criteria of the EWGSOP2, depends on the nutritional status according to BMI in older people, with obese individuals being at greater risk of suffering from sarcopenia. Nearly 50% had probable sarcopenia, a condition that could be detected early with the purpose of taking preventive measures, such a nutritional approach.

## 1. Introduction

Sarcopenia is considered a natural biological process associated with ageing and older people, however, it can also occur earlier in life [1,2]. Some of the factors contributing to sarcopenia are age-related muscle loss, associated diseases, physical inactivity, sedentary behavior, inadequate diets, malnutrition and lifestyles [1,2,3]. It is related to a decrease in muscle strength and muscle mass, poorer physical performance, functional and mobility limitations, greater risk of falls and fractures, cardiovascular and respiratory diseases, and a decline in the quality of life and sensory, cognitive and immune functions [4]. It is a highly prevalent disorder and increases with age [5]. Its prevention and optimal treatment could prevent personal, social and economic burdens [1,6,7].

International studies indicate a prevalence of sarcopenia ranging from 5% to 50% [8,9]. In Chile, background information about its prevalence is limited and varies depending on the criteria and cut-off points used for its detection [9]. In people over 60 years of age the prevalence is 19%, and 30% among people aged 80 years and older, according to the algorithm proposed by the European Working Group of Sarcopenia in Older People (EWGSOP1) [10].

In 2018, the EWGSOP2 updated the algorithm and defined sarcopenia as “a progressive and generalized skeletal muscle disorder that is associated with increased likelihood of adverse outcomes including falls, fractures, physical disability and mortality, and may be acute or chronic” [1]. Unlike the EWGSOP1, these new guidelines use low muscle strength as a primary parameter, since it is a more reliable measure of muscle function than muscle mass when predicting adverse outcomes [1]. 

Furthermore, the new consensus recommends simple measures and cut-off points that define and characterize sarcopenia using the three predictors. In the case of muscle strength assessment, it has been recommended the measurement of grip strength using a dynamometer [1,11]. In addition, the measurement of calf circumference (CC) is considered a simple and inexpensive method, and a good predictor and replacement proxy for the diagnosis of muscle mass in older people [1]. With regard to the gait speed test, it is considered quick, safe, highly reliable and is widely used in the daily practice [1,12]. 

Body Mass Index (BMI) is the most commonly used index to diagnose nutritional status, however, the Mini Nutritional Assessment (MNA) is a validated instrument and a useful mean to assess nutritional status in older people [13]. Studies have shown that 18% of people aged over 60 years of age have an abnormal nutritional status. In Chile, the NHS 2016–2017 reported 78.3% of malnutrition by excess and by deficit among adults aged 65 years and older (according to the BMI cut-off points for adult population; however, this is not in accordance with the BMI ranges proposed for older people) [13,14]. 

It was not possible to find national up-to-date studies on the prevalence of sarcopenia according to the EWGSOP2 algorithm and its relationship with nutritional status in older people. In the light of the above, the objective of this study is to describe and associate sarcopenia and nutritional status in a group of Chilean older adults aged 65 years and older during 2018, using the diagnostic criteria for sarcopenia proposed by the EWGSOP2.

## 2. Materials and Methods

Design and subjects: Descriptive, associative and comparative study, with a cross-sectional design and a quanti-qualitative approach. The present study constitutes a subsample of Chilean older people from the Multicentric Study on Nutrition of Older Adults, 2018.

A number of 171 institutionalized (residents of geriatric nursing homes: assisted living facilities or long-term care settings for older adults, managed according to Chilean social policies) and community-dwelling (subjects who are part of some senior citizens’ club, neighborhood committee or private groups) Chilean older people participated. The study included men and women aged 65 years or older, who were able to perform daily activities without restrictions caused by some health problem, and who agreed to participate in the evaluations the day of the intervention by signing a consent form. The subjects who suffered from a psychological or physical disorder that prevented them from responding the survey or performing the activities included in the study were excluded; the aforementioned information was provided by the caretakers or by the participants themselves. Researchers used a convenience sample and, considering exclusion criteria, the sample finally consisted of 155 older people. 

For the purposes of this study, an older person was defined as a person aged 65 years or older, according to the criterion of the Ministry of Health (MINSAL, for its Spanish acronym) of Chile. Dependent variables were sarcopenia, muscle strength (measured by grip strength using a dynamometer), muscle quantity (assessed by CC), physical performance (assessed by gait speed); the independent variable was nutritional status, assessed by means of BMI and MNA.

Data Collection: data was collected between August and December of the year 2018, by Nutritionists of the Faculty of Health Sciences of the Universidad de Playa Ancha. The assessment and recording of data occurred in a previously monitored place, adapted by the professionals. Muscle strength test was conducted first, using a dynamometer to measure grip strength; afterwards, the MNA was applied as an interview; subsequently, anthropometric measurements were performed to assess weight in kilograms (kg), height in meters (m), brachial circumference and calf circumference in centimeters (cm); finally, gait speed test was the last one to be conducted. All the measurements were directly and immediately recorded in the MNA logbook registers in paper form, prepared by the nutritionists for the purposes of this study.

Diagnosis of sarcopenia: The diagnosis was made using the algorithm of the EWGSOP2, on the basis of 3 criteria: (1) low muscle strength, measured by grip strength; (2) low muscle quantity or muscle mass, measured by CC; (3) low physical performance, assessed by the gait speed test [1]. Probable sarcopenia was identified with criterion 1; the diagnostic of sarcopenia was confirmed by criteria 1 and 2; and severe sarcopenia was found when all 3 criteria were met [1].

Muscle strength: It was assessed by the measurement of grip strength using a JAMAR^®^ hydraulic handheld dynamometer, which measures from 0 to 200 lbs or 90 kg and has 5 grip positions. The technique for measuring strength considered the subject seated in a chair with the back against the backrest, shoulders adducted with no rotation, elbow flexed at 90°, forearm and wrist in a neutral position, feet flat on the floor, the evaluated arm should not be resting on any surface, the dynamometer must be placed vertically and the second position should be used; maximum grip strength was measured for 3 seconds, resting 1 minute between each repetition, performing 2 attempts [15,16]. The dominant hand was used [17,18]. The best measurement of each attempt was used for this study [16]. Outcomes of <27 kg in men and <15 kg in women were considered as low muscle strength; these values were previously determined in studies in Chilean older adults, and were applicable when this study was conducted [5,9,18].

Muscle quantity (or muscle mass): Muscle mass was assessed by the measurement of CC, using a Cescorf flexible anthropometric steel tape; the bottom side of the blade was blank and the tape had a length of 200 cm. The subject was instructed to uncover their calf and remain in anatomical position with their weight evenly distributed on both feet. CC was measured by manipulating the tape in a series of up and down horizontal measurements so as to identify the maximum girth; the measurement was made from the lateral aspect of the leg [19]. A measurement of <31 cm was considered the cut-off value for low muscle quantity in both sexes [1].

Physical performance: The test was conducted in a setting previously measured and implemented for the subject to perform a walking test, and an electronic device was used. The subject was asked to walk a distance of 4 m, while researchers measured the time required to complete the distance [1]. A speed of ≤0.8 m/s was considered low physical performance for both sexes [1].

Nutritional status assessment according to BMI: BMI was assessed by weight measurement using an Omron bioimpedance scale model HBF-514C, with a capacity of 150.0 kg. The technique consisted of instructing the subject to step on the scale (which had been previously calibrated in zero) with no shoes and wearing the least amount of clothes possible, keeping an upright posture and relaxed shoulders, with arms hanging at the sides of the body [19]. Height was measured using the SECA 214 mobile stadiometer, with a measuring range of 20–205 cm. The subject was instructed to step on the stadiometer, standing still on the center of the platform, with an upright position, feet together and arms hanging at the sides of the body. Women were requested not to wear hair clips or bands and to keep their hair down. The measurement was conducted following the Frankfort plane technique [19]. Data reading was recorded before the subject exhaled. Nutritional status was assessed using the BMI (kg/m^2^) for older adults, whose classification corresponded to: underweight (<23), normal nutritional status (23.0–27.9), overweight (28.0–31.9), and obese (≥32), according to the parameters of the MINSAL of Chile [20].

Nutritional status assessment according to the MNA: It was applied as an interview, in parallel with anthropometric measurements of weight, height, calf circumference and brachial circumference. For brachial circumference, the measurement was conducted by placing the measuring tape at the level of the mid-acromiale-radiale line of the non-dominant arm of the subject [19]. Then, the circumference was measured at the acromiale-radiale midpoint, with the arm relaxed and hanging by the sides. Nutritional status was classified according to the following scores: malnutrition (<17 points), risk of malnutrition (17–23.5 points), and normal (25–30 points), with a maximum of 30 points according to the overall assessment [21]. 

Statistical analysis: Numeric variables were expressed as mean, standard deviation. Categorical variables were presented as absolute and relative frequencies (percentage). Results were also described by age group and by place of residence (institutionalized or community-dwelling subjects). The Student’s t test was used to determine the differences by sex, with respect to the mean of muscle strength, CC, physical performance, BMI and global MNA score. The Chi-square test or the Fisher test were used to assess the association between sex and categorical variables such as sarcopenia, muscle strength, muscle quantity, physical performance, and nutritional status (BMI and MNA); the association between age group and nutritional status (BMI and MNA); and the relationship between sarcopenia and nutritional status according to BMI and the MNA. In order to conduct the association tests, two categories were considered for sarcopenia: absence of sarcopenia and sarcopenia, which included probable sarcopenia and severe sarcopenia; however, it did not include sarcopenia with criteria 1 and 2, since none of the participants presented this diagnosis. In the case of the MNA, two categories were identified: normal and malnutrition, which corresponded to risk of malnutrition and malnutrition. *p* values of <0.05 were considered as statistically significant. Analyses were performed using the statistical package STATA v.15 (2017) and the Statistical Package for Social Science (SPSS) software. Finally, a multivariate logistic regression analysis, with adjustments by age and sex was conducted with the purpose of identifying the association between sarcopenia and nutritional status according to BMI. Sarcopenia was categorized as 0 for individuals without sarcopenia, and 1 for individuals with some degree of sarcopenia. The final regression model was developed through the stepwise procedure, keeping in the final model those variables with an association probability of 0.10 (sex and age). Odds ratio and 95% confidence interval were used to determine the magnitude of the association. Significance level was set at *p* < 0.05. Regression analyses were conducted with the Stata 17 software (StataCorp, College Station, TX, USA).

## 3. Results

General characteristics of the sample: A total of 155 older adults participated in the study, 72.3% of whom were women. Average age was 77.1 ± 7.1, with men having greater weight and height with respect to women (both <0.01) (Table 1).

Results according to place of residence are shown in Table 2. The 67.7% of the participants consisted of non-institutionalized subjects, who had greater BMI and calf circumference values, but less muscle strength, with respect to institutionalized subjects (all *p* < 0.01).

Diagnosis of sarcopenia: A percentage of 49.7%, 45.2%, and 5.2% of the sampled subjects had probable sarcopenia, absence of sarcopenia, and severe sarcopenia, respectively. No association between sarcopenia and sex (*p* = 0.83) was found; however, no significant associations showed that women had a higher prevalence of probable sarcopenia (50.9%) when compared to men (46.5%), and a lower prevalence of severe sarcopenia (4.5%, 7%, respectively) (Table 3). In addition, the presence of sarcopenia depended on the place of residence (*p* = 0.04), with the highest occurrence of probable sarcopenia being found among non-institutionalized subjects (55.2%) (Table 2).

Muscle strength: The mean muscle strength in the total sample was 17.3 ± 8.6 (Table 1). A percentage of 54.8% of the subjects presented low muscle strength (Table 4). Significant differences were found between men and women (*p* < 0.01) (Table 1), nevertheless, no association was found between sex and the categorized variable muscle strength (*p* = 0.83).

Muscle quantity (or muscle mass): Mean CC was 35.1 ± 3.4, no significant differences were found between men and women (*p* = 0.68) (Table 1); 7.1% of the subjects had low muscle quantity When categorizing muscle quantity, there was also no association with sex (*p* = 1.00) (Table 4).

Physical performance: Mean gait speed was 0.6 ± 0.3. No significant differences were found between men and women (*p* = 0.90) (Table 1); 85.2% of the subjects showed low physical performance. No significant differences were found between men and women (*p* = 0.90) (Table 1). When categorizing physical performance, there was no association with sex (*p* = 0.76) (Table 4).

Nutritional status according to MNA: The mean of the global assessment was 24.5 ± 3.3. No significant differences were found in the global MNA between men and women (*p* = 0.81) (Table 1). With respect to nutritional status according to the MNA, 29% of the subjects presented risk of malnutrition, and 4.5% presented malnutrition (Table 1).

Nutritional status according to BMI: The mean BMI was 29.1 ± 5.2 and no significant BMI differences were found between men and women (*p* = 0.08) (Table 1). The 52.9% of the subjects had malnutrition by excess (overweight and obesity), and 7.1% was underweight. The prevalence of underweight was 3% higher in men when compared to women, while women presented a higher prevalence for overweight (26.8%) and obesity (30.4%) in comparison with men (16.3% and 25.6%, respectively); however, there was no association between sex and nutritional status according to BMI (*p* = 0.36) (Table 1).

Association between nutritional status and sarcopenia: Bivariate analyses showed that participants with sarcopenia (probable and severe) and without sarcopenia had a homogeneous behavior regarding their distribution according to the MNA (*p* = 0.39). Conversely, nutritional status was associated with sarcopenia, according to BMI (*p* = 0.02). According to sex, no association was found between nutritional status and sarcopenia according to the MNA, neither in men (*p* = 0.99) nor in women (*p* = 0.31); moreover, no association was found between nutritional status according to BMI and sarcopenia in men (*p* = 0.31), but an association was found in women (*p* = 0.03) (Table 5).

Multivariate analysis found an association between sarcopenia and nutritional status according to BMI, when adjusting by sex and age, showing that participants with obesity had 3.2 times more risk of suffering from sarcopenia, when compared to participants with normal nutritional status (OR: 3.20; 95% CI: 1.24; 8.26), and subjects with undernutrition showed 7.8 times more risk of sarcopenia, however, this association was not significant (OR: 7.82; 95% CI: 0.91; 67.21) (Table 6).

## 4. Discussion

The present study assessed sarcopenia and its association with nutritional status in a group of Chilean older adults aged 65 years and older, using the diagnostic criteria for sarcopenia proposed by the EWGSOP2. In this study, the prevalence of probable sarcopenia was of 49.7%. The outcomes obtained in this group of older people, show that there is an association between nutritional status and sarcopenia according to BMI. More interestingly, older adults with obesity had more risk of suffering from sarcopenia when compared to subjects with a normal nutritional status. This is one of the first studies in Chile to use the EWGSOP2 criteria for the diagnosis of sarcopenia.

Overall estimates of prevalence at a global level indicate that 10% of both men and women suffer from sarcopenia [22]. In Chile, the prevalence of sarcopenia in a cross-sectional sample (2016) of 1006 subjects older than 60 years was of 19.1%, a similar percentage among men and women, reaching nearly 40% in people aged 80 and older when the EWGSOP 2010 algorithm was used [10]. Another study that collected data between 2012 and 2017 in a population whose average age was 68.2 years old, showed a prevalence of pre-sarcopenia of 18.4%, and a prevalence of sarcopenia of 24.2%, with the prevalence being higher in men than in women according to the criteria of the EWGSOP 2010 [5]. In our study, nearly 50% of the sample had probable sarcopenia, that is, low muscle strength, with criterion 1 being met; however, none of the subjects met only criteria 1 and 2, that is, low muscle strength and low muscle mass, necessary to confirm the diagnosis of sarcopenia. Nevertheless, 5.2% had severe sarcopenia, meeting all 3 criteria. No significant differences were observed between both sexes in the group of severe sarcopenia. It was noted that sarcopenia becomes worse after turning 75 years old and remains with no changes after 80 years old; these outcomes cannot be extrapolated with the national evidence available, since the assessment was conducted using the new guidelines of the EWGSOP2.

Regarding the criteria of the EWGSOP2, the JAMAR^®^ dynamometer has been used for the evaluation of muscle strength; this is a validated and widely used instrument, however, it requires data from appropriate reference populations [1]. It is a common, easy and reproducible method, that does not require the intervention of the examiner and has adjustable handles with five different positions, with the second position being the most recommended to perform the assessment of maximum muscle strength [15]. Evidence shows that muscle strength begins to decline gradually after turning 50 years old [15]. In Chile, there are few studies addressing grip strength measurement [15]. Researchers have proposed cut-off points, and prevalence rates of low muscle strength of 60% and 63% have been observed in men and women, respectively, according to the EWGSOP 2010 algorithm. Mean strength is significantly higher in men as compared to women of all the ages; every year, a person loses 0.2 kg of strength. In our country there are previously validated cut-off points (<27 kg men; <15 kg women) [9]. Between 2016 and 2018, it was conducted a study including 901 Chilean people aged 20–70 years, which showed that mean strength in men is significantly higher than in women of all ages (*p* < 0.01). Strength measurements obtained from women showed a strength of 17 kg less than that of men, with adjustments by age; strength declines 0.2 kg for every year of age, with adjustments by sex [15]. These results are in line with the outcomes of this study, which noted significant differences between sexes (*p* < 0.01), indicating that the mean strength in men is significantly higher as compared to women (25.4 kg and 14.2 kg, respectively).

With respect to the assessment of muscle quantity, the dual-energy X-ray absorptiometry (DEXA) is considered the gold standard for the measurement of muscle mass, nevertheless, is expensive and not readily accessible [9,12]. For this reason, it is necessary to implement widely available and cost-effective screening tools in the primary healthcare centers [5]. The MINSAL of Chile recommends the use of anthropometric measurements as the method of choice over the DEXA for people aged 50 years or older [23]. This study uses CC as an indicator of low muscle quantity, following the indications of the EWGSOP 2010 and EWGSOP2 consensus. Anthropometry might not be an appropriate method for assessing muscle mass, despite being a simple and a strong predictor of future diseases, functional impairment and mortality [1,24]. It has been demonstrated that CC predicts performance and survival in older people, thus, CC measurements may be used as a diagnostic proxy in settings where no other muscle mass diagnostic methods are available [1]. In a clinical practice context, 60% of physicians from different countries used CC as a tool for the assessment of muscle mass [12]. A study conducted in five cities of Latin America indicated an average CC of 36.1 in men and 35.2 in women in Chile, with greater CC measurements in men (T-test, *p* < 0.01) [25]. Our study noted an average CC of 35.2 ± 3.2 and 35.0 ± 3.5 cm in men and women, respectively, without showing significant differences between sexes (*p* < 0.68).

Physical performance has been assessed using the gait speed test and other alternatives such as the chair stand test (5-times sit-to-stand) [1,5]. Chilean studies have considered a distance of 3 meters, with an average of 0.9 m/s ± 0.3 being observed between 2012 and 2013, with no significant differences in both sexes; and an average of low physical performance of 0.9 ± 0.2 according to the criteria of the EWGSOP 2010 [5,9]. An average of 0.6 ± 0.3 m/s was observed in our study, with low physical performance being observed, with an average of 0.56 ± 0.2 m/s with no significant differences between both sexes (*p* = 0.90). Literature has reported that at the age of 60, 15% of the subjects show gait alterations; at the age of 70 the percentage reaches 35% and increases up to 50% in people over 85 years old [26].

The MNA is considered the gold standard for nutritional assessment, because it is inexpensive, reliable, non-invasive and easy to use, although it is a limited tool for nutritional detection [27,28]. It is quick to complete and identifies geriatric patients aged 65 years or older who suffer from undernutrition or are at risk of undernutrition [21]. Sarcopenia is directly associated with undernutrition or undernutrition risk, and with age in subjects older than 65 years [1,10,29]. In the present study, 7.1% of the sample is within the range of underweight according to their BMI (9.3% men, 6.3% women). Undernutrition is strongly associated with multimorbidity, premature mortality, poorer quality of life, increased institutionalization, and greater need for medical care, fluctuating from 1.3% to 47.8% among people aged 60 years or older [30]. On the other hand, in a sample of Korean elderly population older than 60 years, 1.3% had sarcopenic obesity, as well as 0.6% of adults over 65 years of age of the Tianliao District [13]. This research found that 53% of the sample are overweight or obese, according to the BMI, demonstrating that obese older adults had more risk of suffering from sarcopenia than individuals with normal nutritional status. Some authors have suggested that obesity itself might be a determining factor in muscle mass loss, because of metabolic disorders associated with this disease, such as insulin resistance, systemic oxidative stress mainly affecting skeletal muscle mass, and inflammation [31].

This study had some limitations, with the most significant being sample size and type of sampling method, which consisted of a non-probabilistic, convenience sample, therefore, results might not be extrapolated to the overall population. However, the sample was constituted by a high percentage of non-institutionalized older people of a medium-low income level, which indeed could be representative of a great number of the population of Chilean older adults. Secondly, this study employed the measurement of CC for the assessment of muscle mass. Nevertheless, in Chile there are validated equations for the Chilean population that were not included in this study, such as muscle mass predictors which are highly consistent with the results determined by the DEXA; these instruments include anthropometric variables such as knee height, weight, calf circumference, hip circumference, dynamometry, sex and age [9,29]. However, the EWGSOP2 considers CC useful as a diagnostic proxy for muscle mass in settings where no other methods are available. Finally, with respect to the diagnosis of undernutrition assessed using BMI, this technique could present some limitations, since this parameter measures a person’s weight in relation to their height, and does not consider body composition, therefore, undernutrition could be underdiagnosed in our sample.

## 5. Conclusions

These results confirm the existence of an association between nutritional status and sarcopenia according to BMI, and that obese older adults had more risk of sarcopenia than subjects with a normal nutritional status. Moreover, almost 50% of the subjects had probable sarcopenia, a condition that could be detected early in older people, with the purpose of taking preventive measures in the primary healthcare network.

The algorithm of the EWGSOP2 could be considered for the diagnosis of sarcopenia, updating the methods available at national level, such as the software tool of the MINSAL; it is recommended to consider anthropometric measurements and prediction equations over the DEXA, since they are readily accessible, cost-effective, and would allow a greater accessibility to sarcopenia research. It would be interesting to assess sarcopenia in people younger than 65 years old, including subjects from different socio-economic levels, in order to visualize its impact in aspects such as health, economy and life expectancy of the Chilean population.

## Figures and Tables

**Table 1:** Characteristics of the sample according to sex.

Variables	Total Sample *n* = 155 (100)	Men *n* = 43 (27.7)	Women *n* = 112 (72.3)	*p* Value
Age (years)	77.1 ± 7.1	76.5 ± 7.1	77.4 ± 7.1	0.24 ^(1)^
Weight (kg)	68.8 ± 13.7	74.7 ± 13.6	66.6 ± 13.1	<0.01 ^(1)^
Height (m)	1.53 ± 0.1	1.63 ± 0.1	1.49 ± 0.1	<0.01 ^(1)^
BMI (kg/m^2^)	29.1 ± 5.2	27.9 ± 4.8	29.6 ± 5.3	0.08 ^(1)^
Nutritional status (BMI)				
Underweight	11 (7.1)	4 (9.3)	7 (6.3)	0.36 ^(2)^
Normal	62 (40)	21 (48.8)	41 (36.6)	
Overweight	45 (29)	7 (16.3)	30 (26.8)	
Obese	37 (23)	11 (25.6)	34 (30.4)	
MNA (score)	24.5 ± 3.3	24.4 ± 3.3	24.5 ± 3.4	0.81 ^(1)^
Nutritional status (MNA)				
Normal	103 (66.5)	28 (65.1)	75 (67)	0.66 ^(2)^
Risk of malnutrition	45 (29)	12 (27.9)	33 (29.5)	
Malnutrition	7 (4.5)	3 (7)	4 (3.6)	
Brachial circumference (cm)	28.5 ± 3.9	28.2 ± 3.7	28.7 ± 3.9	0.25 ^(1)^
Muscle strength (kg)	17.3 ± 8.6	25.4 ± 9.7	14.2 ± 5.6	<0.01 ^(1)^
Calf circumference (cm)	35.1 ± 3.4	35.2 ± 3.2	35.0 ± 3.5	0.68 ^(1)^
Gait speed (m/s)	0.6 ± 0.3	0.6 ± 0.3	0.6 ± 0.3	0.9 ^(1)^

BMI: Body Mass Index (kg/m^2^) of the older person: MNA: Mini Nutritional Assessment. Values are expressed as mean and standard deviation. *p* value equals to: ^(1)^: Student’s *t*-test. ^(2)^: Chi-square test.

**Table 2:** Characteristics of the sample according to type of residence.

	Total Sample *n* = 155 (100)	Non-Institutionalized *n* = 105 (67.7)	Institutionalized *n* = 50 (32.3)	*p* Value
Age				
Average (*SD*)	77.1 (7.1)	77 (6.2)	77.4 (8.9)	0.76 ^(2)^
Median (Q1–Q3)	77 (72–82)	77 (72–80)	75 (71–83)	
Weight				
Average (*SD*)	68.8 (13.7)	69.6 (13.3)	67.3 (14.7)	0.41 ^(2)^
Median (Q1–Q3)	68.5 (59.7- 75.6)	68.6 (60.3–75.6)	67.8 (56.5–75)	
Height				
Average (*SD*)	1.54 (0.09)	1.52 (0.08)	1.57 (0.10)	<0.01 ^(2)^
Median (Q1–Q3)	1.5 (1.5–1.6)	1.5 (1.5–1.6)	1.6 (1.5–1.6)	
BMI				
Average (*SD*)	29.1 (5.2)	30.1 (5.2)	27.2 (4.7)	<0.01 ^(2)^
Median (Q1–Q3)	28.3 (25.8–31.7)	29.3 (26.5–32.5)	26.8 (24–30.3)	
Nutritional status (BMI)				
Normal	62 (40%)	36 (34.3%)	26 (52%)	0.02 ^(1)^
Underweight	11 (7.1%)	5 (4.8%)	6 (12%)	
Overweight	45 (29%)	33 (31.4%)	12 (24%)	
Obese	37 (23.9%)	31 (29.5%)	6 (12%)	
MNA (score)				
Average (*SD*)	24.5 (3.4)	24.8 (3.1)	23.9 (3.8)	0.24 ^(2)^
Median (Q1–Q3)	25 (22.5–27)	25.5 (23–227)	25 (21.5–27)	
Nutritional status (MNA)				
Normal	103 (66.5%)	74 (70.5%)	29 (58%)	0.05 ^(1)^
Risk of malnutrition	45 (29%)	29 (27.6%)	16 (32%)	
Malnutrition	7 (4.5%)	2 (1.9%)	5 (10%)	
Brachial circumference				
Average (*SD*)	28.5 (3.9)	28.9 (3.9)	27.7 (3.9)	0.08 ^(2)^
Median (Q1–Q3)	28 (26–31.5)	28 (26–32)	27 (25–30)	
Muscle strength				
Average (*SD*)	17.3 (8.6)	15.6 (6.7)	21.1 (10.6)	<0.01 ^(2)^
Median (Q1–Q3)	16 (12–22)	14.0 (12–20)	19 (12–28)	
Calf circumference				
Average (*SD*)	35.1 (3.4)	35.6 (3.4)	33.94 (3.17)	<0.01 ^(2)^
Median (Q1–Q3)	34.5 (32.5–37.1)	35.5 (33–38)	33.3 (32–36)	
Gait speed				
Average (*SD*)	0.65 (0.28)	0.67 (0.26)	0.6 (0.3)	0.16 ^(2)^
Median (Q1–Q3)	0.7 (0.4–0.8)	0.7 (0.5–0.8)	0.6 (0.4–0.8)	
Sarcopenia				
Absence of sarcopenia	70 (45.2%)	44 (41.9%)	26 (52%)	0.04 ^(1)^
Severe sarcopenia	8 (5.2%)	3 (2.9%)	5 (10%)	
Probable sarcopenia	77 (49.7%)	58 (55.2%)	19 (38%)	

BMI: Body Mass Index (kg/m^2^) of the older person: MNA: Mini Nutritional Assessment. *p* value equals to: ^(1)^: Chi-square test. ^(2)^: Wilcoxon’s test.

**Table 3:** Diagnosis of sarcopenia according to the EWGSOP2 criteria, by sex.

Variables	Total Sample *n* = 155 (100)	Men *n* = 43 (27.7)	Women *n* = 112 (72.3)	*p* Value
Absence of sarcopenia	70 (45.2)	20 (46.5)	50 (44.6)	0.83
Probable sarcopenia	77 (49.7)	20 (46.5)	57 (50.9)	
Severe sarcopenia	8 (5.2)	3 (7)	5 (4.5)	

Values are expressed as number of subjects (percentage). *p* value equals to Chi-square test.

**Table 4:** Muscle strength, muscle quantity/quality and physical performance according to sex.

Variables	Total Sample *n* = 155 (100)	Men *n* = 43 (27.7)	Women *n* = 112 (72.3)	*p* Value
Muscle strength				
Normal	70 (45.2)	20 (46.5)	50 (44.6)	0.83 ^(1)^
Low	85 (54.8)	23 (53.5)	62 (55.4)	
Muscle quantity				
Normal	144 (92.9)	40 (93)	104 (92.9)	1.00 ^(2)^
Low	11 (7.1)	3 (7)	8 (7.1)	
Physical performance				
Normal	23 (14.8)	7 (16.3)	16 (14.3)	0.76 ^(1)^
Low	132 (85.2)	36 (83.7)	96 (85.7)	

Values are expressed as number of subjects (percentage). *p* value equals to: ^(1)^: Chi-square test. ^(2)^: Fisher’s exact test.

**Table 5:** Association between sarcopenia and nutritional status according to Body Mass Index and Mini Nutritional Assessment.

Nutritional Status	Total Sample *n* = 155 (100)	Absence of Sarcopenia *n* = 70 (45.2)	Probable Sarcopenia + Severe Sarcopenia *n* = 85 (54.8)	*p* Value
BMI				0.02
Underweight	11 (7.1)	1 (0.6)	10 (6.5)	
Normal	62 (40)	30 (19.4)	32 (20.6)	
Overweight	37 (23.9)	13 (8.4)	24 (15.5)	
Obese	45 (29)	26 (16.8)	19 (12.3)	
MNA				0.39
Normal	103 (66.5)	49 (31.6)	54 (34.8)	
Risk of Malnutrition + Malnutrition	52 (33.5)	21 (13.5)	31 (20)	

BMI: Body Mass Index (kg/m^2^) of the older person: MNA: Mini Nutritional Assessment. Values are expressed as number of subjects (percentage). *p* value equals to Chi-square test.

**Table 6:** Association between sarcopenia and nutritional status, according to Body Mass Index.

Nutritional Status	*n*	OR 95% CI	*p* value
Underweight	11	7.82 (0.91; 67.21)	0.061
Normal	62	1.00 (Ref)	
Overweight	45	0.81 (0.35; 1.89)	0.631
Obese	37	3.20 (1.24; 8.26)	0.016

## Data Availability

The data presented in this study is available on request from the corresponding author. The data are not publicly available because they contain personal health information.
